# Development of cellulose nanocrystal-stabilized Pickering emulsions of massoia and nutmeg essential oils for the control of *Aedes albopictus*

**DOI:** 10.1038/s41598-021-91442-6

**Published:** 2021-06-08

**Authors:** Seon-Mi Seo, Jae-Woo Lee, Jonghyun Shin, Jun-Hyung Tak, Jinho Hyun, Il-Kwon Park

**Affiliations:** 1 Department of Agriculture, Forestry and Bioresources, College of Agriculture and Life Sciences, Seoul National University, Seoul, 08826 Republic of Korea; 2 Research Institute of Agriculture and Life Science, College of Agriculture and Life Sciences, Seoul National University, Seoul, 08826 Republic of Korea; 3 Department of Agricultural Biotechnology, College of Agriculture and Life Sciences, Seoul National University, Seoul, 08826 Republic of Korea

**Keywords:** Entomology, Biomaterials

## Abstract

We investigated the larvicidal potential of 10 plant essential oils (EOs) against the Asian tiger mosquito *Aedes albopictus*. Among the EOs, larvicidal activity against *Ae. albopictus* was strongest in those derived from massoia (*Massoia aromatica*) and nutmeg (*Myristica fragrans*). Larvicidal activities of massoia and nutmeg EOs against *Ae. albopictus* were 95.0% and 85.0% at 50 μg/mL, respectively. A total of 4 and 14 compounds were identified from massoia and nutmeg, respectively, and two massoia lactones, C10 and C12, were isolated from massoia EO. Among the identified compounds, benzyl salicylate, terpinolene, C12 massoia lactone, sabinene, benzyl benzoate, methyl eugenol, and C10 massoia lactone exhibited the strong larvicidal activity. Cellulose nanocrystal (CNC)-stabilized Pickering emulsions of massoia and nutmeg EOs were developed to overcome the insolubility of EOs in water. CNC/massoia and CNC/nutmeg emulsions were stable for at least 10 days, and larvicidal activities of CNC/massoia PE and CNC/nutmeg were higher than those of crude massoia and nutmeg EOs. This study presents a CNC-stabilized PE, a suitable formulation for EOs, as a potential larvicide against *Ae. albopictus.*

## Introduction

The Asian tiger mosquito *Aedes albopictus* transmits the dengue virus and chikungunya virus, both of which cause human diseases^1^. *Aedes albopictus* is also an insect vector of the Zika virus, which causes microcephaly and other congenital abnormalities in developing fetuses and newborns^2^. Although *Ae. albopictus* is native to East Asia, it has spread to Europe, North and South America, the Middle East, and Africa^1^. Insect growth regulators (e.g. methoprene, novaluron or pyriproxyfen) and temephos are widely used to control *Ae. albopictus* larvae, but selection for resistance to some of current larvicides has been documented around the world^1,3–6^, creating a need for new control agents.

Essential oils (EOs) have attracted attention for their potential medicinal applications^7–9^. Besides medicinal uses, many researchers have become interested in the insecticidal and repellent activities of EOs^10–13^. Larvicidal activity against mosquitoes such as *Ae. albopictus* and *Ae. aegypti* has been documented in many EOs, including *Citrus* and *Cryptomeria japonica* EOs^14–18^. However, many studies have not figured out the active components of EOs which have shown good activities against the mosquitos, despite the importance of the analysis. As to the physicochemical properties of EOs, high hydrophobicity makes it difficult to develop EOs that can serve as larvicides against mosquitoes. To overcome the insolubility of EOs in water, proper formulation is essential^19^. Emulsions are commonly used in the pesticide industry to incorporate hydrophobic materials into aqueous products^20^. However, synthetic and organic phase emulsions are associated with multiple undesirable side effects^20^. Recently, Pickering emulsions (PEs) stabilized by solid particles, and cellulose nanocrystals (CNCs) in particular, are being considered because of their low environmentally impact, bio-degradability, and low production cost^21^.

Here, we describe the larvicidal potential of 10 EOs against the third instar stage of *Ae. albopictus* to identify new and effective larvicides and analyzed the chemical composition of the two most promising ones (i.e., massoia and nutmeg EOs). Furthermore, the toxicity of the individual components of massoia (*Massoia aromatica*) and nutmeg (*Myristica fragrans*) EOs is described. Finally, formulations based on CNC-stabilized PEs were developed, and their larvicidal activities were compared with those of crude EOs.

## Results

### Larvicidal activities of Eos

The larvicidal activities of 10 EOs against *Ae. albopictus* are shown in Table 1. Larvicidal activities of massoia and nutmeg EOs were greater than those of other EOs. Larvicidal activities of massoia and nutmeg EOs were 95.0% and 85.0% at 50 μg/mL, respectively, but reduced to 37.5% and 12.5% at 25 μg/mL, respectively. Other EOs showed less than 40% larvicidal activity against *Ae. albopictus* at 50 μg/mL.

**Table 1:** Larvicidal activity of 10 EOs against *Ae. albopictus*.

Plant EOs	Plant species	Origin	Batch No	Mortality (%, Mean ± SE^b^, N^c^ = 4)		
				Concentration (μg/mL)		
				100	50	25
Blue cypress	*Calitis intratropica*	Australia	NA	42.5 ± 4.8de^d^	-	-
Hinoki	*Chamaecyparis obtusa*	Japan	15668–110689	12.5 ± 2.5f	-	-
Texas cedar	*Juniperus mexicana*	USA	3100–10193	57.5 ± 2.5bcd	32.5 ± 2.5b	2.5 ± 2.5b
Japanese cedar	*Cryptomeria japonica*	Reunion	32–135148	75.0 ± 6.5abc	7.5 ± 4.8bc	-
Fir needle	*Abies holophylla*	NA^a^	NA	60 ± 4.1bcd	17.5 ± 2.5bc	-
Sprice, spruce	*Picea mariana*	NA	NA	47.5 ± 4.8cde	-	-
Golden lotus	*Nymphaea mexicana*	Thailand	19948–109767	85.0 ± 5.0ab	20.0 ± 4.1bc	-
Cubeb	*Piper cubeba*	Indonesia	128610–78475	20.0 ± 8.2ef	-	-
Massoia	*Massoia aromatica*	Indonesia	206522–101257	100a	95.0 ± 2.9a	37.5 ± 4.8a
Nutmeg	*Myristica fragrans*	NA	NA	90.0 ± 4.1a	85.0 ± 9.6a	12.5 ± 2.5b
Control				0f	0c	0b
				F _10,33_ = 52.94	F _6,21_ = 65.95	F _3,12_ = 33.12
				*P* < 0.0001	*P* < 0.0001	*P* < 0.0001

^a^Not available; ^b^Standard error of the mean; ^c^Replication number; ^d^Means within a column followed by the same letters are not significantly different (Scheffe’s test).

### Chemical analysis of massoia and nutmeg Eos

The chemical compositions of massoia and nutmeg EOs are supplied in Table 2. Fourteen compounds were determined from nutmeg EO. The most abundant constituent in nutmeg EO was sabinene (50.44%) followed by *α*-pinene (12.21%), myristicin (7.38%), limonene (6.29%), and terpinen-4-ol (4.08%). All other constituents accounted for less than 4%. Four compounds, including C10 massoia lactone (79.97%), C12 massoia lactone (10.75%), benzyl benzoate (6.55%), and benzyl salicylate (1.13%) were identified in massoia EO.

**Table 2:** Chemical analysis of massoia and nutmeg EOs.

Compounds	Retention index (RI)			Composition (%)	
	DB-5MS	HP INNOWAX	Beta dex 120	Nutmeg	Massoia
*α*-Pinene	930	1021		12.21	–
(−)-*α*-Pinene	(930)	(1021)	1023	(8.75)	–
(+)-*α*-Pinene	(930)	(1021)	1027	(3.46)	–
Sabinene	970	1128		50.44	–
Myrcene	989	1165		1.58	–
*α*-Phellandrene	1003	1165		2.54	–
*α*-Terpinene	1014	1181		1.56	–
Limonene	1027	1200		6.29	–
(-)-Limonene	(1027)	(1200)	1115	(2.2)	–
( +)-Limonene	(1027)	(1200)	1118	(4.09)	–
*γ-*Terpinene	1056	1248		2.63	–
Terpinolene	1082	1287		1.05	–
Terpinen-4-ol	1179	1610		4.08	–
Safrole	1287	1897		1.23	–
Methyl eugenol	1399	2025		0.61	–
Isoeugenol	1445	2330		0.54	–
C10 massoia lactone	1482	2261		–	79.97
Myristicin	1517	2283		7.38	–
Elemicin	1558	2243		3.00	–
C12 massoia lactone	1689	2490		–	10.75
Benzyl benzoate	1762	2647		–	6.55
Benzyl salicylate	1862	2829		–	1.13
Total				95.14	98.40

### Identification of C10 and C12 massoia lactones

C10 and C12 massoia lactones were identified and obtained using bioassay-guided isolation because two compounds were commercially unavailable. The chemical structures of the isolated compounds were confirmed by ^1^H, ^13^C nuclear magnetic resonance (NMR) and mass spectrometer data. The purities of isolated C10 massoia lactone and C12 massoia lactone were 95% and 95%, respectively.

C10 massoia lactone C_10_H_16_O_2_, electron ionization mass spectrometry (EI-MS) m/z: 168, 139, 122, 108, 97, 81, 68, 55, 41. ^1^H NMR (600 MHz, CDCl_3_): δ 0.91 (3H, t, *J* = 7.0, H-10), 1.28–1.35 (4H, m H-8,9), 1.39–1.42 (^1^H, m, H-7a), 1.50–1.53 (^1^H, m, H-7b), 1.61–1.67 (^1^H, m, H-6a), 1.77–1.88 (^1^H, m, H-6b), 2.23–2.34 (2H, m, H-4), 4.40–4.44 (^1^H, m, H-5), 6.02 (^1^H, d, *J* = 9.78, H-2), 6.86–6.98 (^1^H, m, H-3). ^13^C NMR (150 MHz, CDCl_3_): δ 13.98 (C-10), 22.50 (C-9), 24.61 (C-7), 29.32 (C-4), 31.64 (C-8), 34.83 (C-6), 78.04 (C-5), 121.44 (C-2), 145.06 (C-3), 164.64 (C-1).

C12 massoia lactone C_12_H_20_O_2_, electron ionization mass spectrometry (EI-MS) m/z: 196, 178, 136, 111, 97, 81, 68, 55, 41. ^1^H NMR (600 MHz, CDCl_3_): δ 0.91 (3H, t, *J* = 7.0, H-12), 1.27–1.35 (4H, m H-8,9,10,11), 1.39–1.42 (^1^H, m, H-7a), 1.50–1.53 (^1^H, m, H-7b), 1.61–1.67 (^1^H, m, H-6a), 1.77–1.83 (^1^H, m, H-6b), 2.31–2.36 (2H, m, H-4), 4.40–4.44 (^1^H, m, H-5), 6.02 (^1^H, d, *J* = 9.6, H-2), 6.86–6.89 (^1^H, m, H-3). ^13^C NMR (150 MHz, CDCl_3_): δ 13.98 (C-12), 22.62 (C-11), 24.81 (C-7), 29.12 (C-8), 29.32 (C-9), 29.47 (C-4), 31.87 (C-10), 34.84 (C-6), 78.34 (C-5), 121.45 (C-2), 145.04 (C-3), 164.63 (C-1).

### Larvicidal activity of major constituents identified in massoia and nutmeg Eos

The larvicidal activities against *Ae. albopictus* of the constituents derived from massoia and nutmeg EOs are shown in Table 3. Among the identified compounds, benzyl salicylate, C12 massoia lactone, terpinolene, C10 massoia lactone, sabinene, benzyl benzoate, and methyl eugenol showed > 80% larvicidal activity at 25 μg/mL. Larvicidal activities of other compounds were less than 60% at 25 μg/mL. Larvicidal activity of temephos was 100% at all test concentrations (100 to 1.5625 μg/mL).

**Table 3:** Larvicidal activity of components identified in massoia and nutmeg EOs against *Ae. albopictus*.

Test materials	Mortality (%, Mean ± SE^a^, N^b^ = 4)						
	Concentration (μg/mL)						
	100	50	25	12.5	6.25	3.125	1.5625
(-)-*α*-Pinene	0 c^c^	–	–	–	–	–	–
( +)-*α*-Pinene	0c	–	–	–	–	–	–
Sabinene	100a	92.5 ± 4.8ab	85.0 ± 6.5ab	55.0 ± 6.5b	25.0 ± 6.5b	–	–
Myrcene	42.5 ± 4.8b	20.0 ± 4.1 cd	–	–	–	–	–
*α*-Phellandrene	100a	57.5 ± 4.8bc	12.5 ± 2.5ef	–	–	–	–
*α*-Terpinene	100a	55.0 ± 6.5bc	5.0 ± 5.0f	–	–	–	–
(-)-Limonene	100a	65.0 ± 13.2ab	27.5 ± 10.31def	–	–	–	–
( +)-Limonene	100a	70.0 ± 9.1ab	10.0 ± 4.1f	–	–	–	–
*γ*-Terpinene	100a	87.5 ± 6.3ab	30def	–	–	–	–
Terpinolene	100a	100a	97.5 ± 2.5a	90.0 ± 7.1a	65.0 ± 8.7a	42.5 ± 4.8a	22.5 ± 2.5a
Terpinen-4-ol	10c	–	–	–	–	–	–
Safrole	100a	67.5 ± 2.5ab	45.0 ± 5de	2.5 ± 2.5c	–	–	–
Eugenol	100a	87.5 ± 2.5ab	52.5 ± 2.5bcd	25.0 ± 5bc	–	–	–
Methyl eugenol	100a	100a	82.5 ± 2.5abc	42.5 ± 4.8b	2.5 ± 2.5b	–	–
Isoeugenol	100a	85.0 ± 2.9ab	22.5 ± 2.5def	–	–	–	–
Methyl isoeugenol	100a	97.5 ± 2.5a	55.0 ± 6.5bcd	27.5 ± 7.5bc	–	–	–
C10 Massoia lactone	100a	100a	95.0 ± 2.9a	25.0 ± 2.9bc	–	–	–
Myristicin	100a	92.5 ± 2.5ab	50.0 ± 4.1 cd	0c	–	–	–
Elemicin	37.5 ± 4.8b	2.5 ± 2.5d	–	–	–	–	–
C12 Massoia lactone	100a	100a	100a	55.0 ± 2.9b	17.5 ± 4.8b	–	–
Benzyl Benzoate	100a	100a	85.0 ± 6.5ab	42.5 ± 7.5b	20.0 ± 4.1b	–	–
Benzyl salicylate	100a	100a	100a	100a	70.0 ± 7.1a	60.0 ± 7.1a	27.5 ± 4.8a
Control	0c	0d	0f	0c	0b	0b	0b
	F _22,69_ = 775.62	F _19,60_ = 47.78	F _17,54_ = 65.38	F _11,36_ = 46.64	F _6,21_ = 26.07	F _2,9_ = 39.17	F _2,9_ = 22.07
	*P* < 0.0001	*P* < 0.0001	*P* < 0.0001	*P* < 0.0001	*P* < 0.0001	*P* < 0.0001	*P* < 0.001

^a^Standard error of the mean; ^b^Replication number; ^c^Means within a column followed by the same letters are not significantly different (Scheffe’s test).

### Physical properties of CNC-based PEs

**Figure 1:**
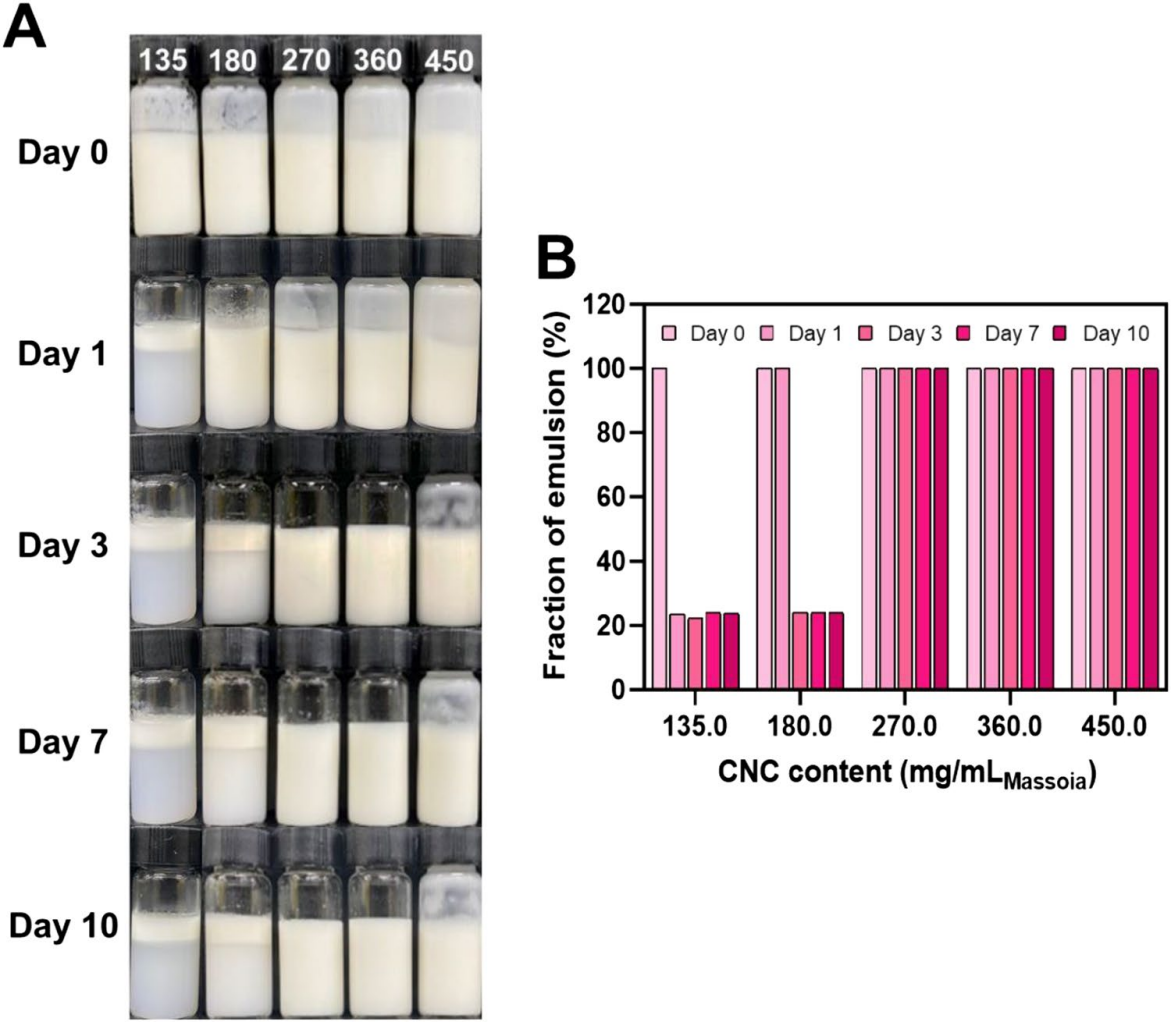
Phase separation of CNC/massoia PE solutions at different CNC content and storage time. (**A**) Digital images of PE solution and (**B**) the fraction of emulsion stored for 10 days.

**Figure 2:**
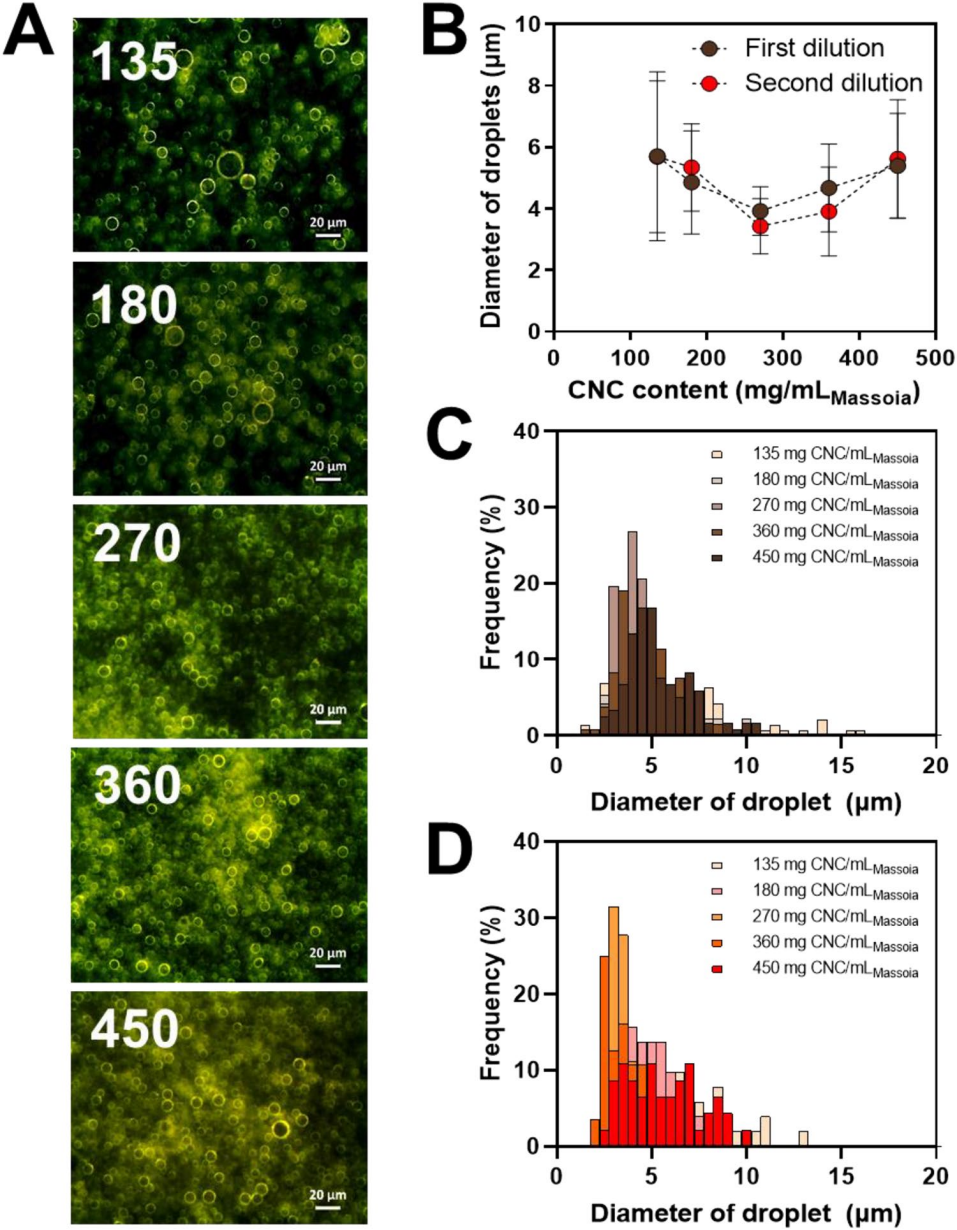
CNC/massoia PEs at different CNC contents. (**A**) Dark-field microscopic images of PE, (**B**) size of emulsion droplets and (**C**,**D**) size distribution of emulsion droplets. (**C**) First dilution and (**D**) second dilution. N = 300, error bar = SD.

**Figure 3:**
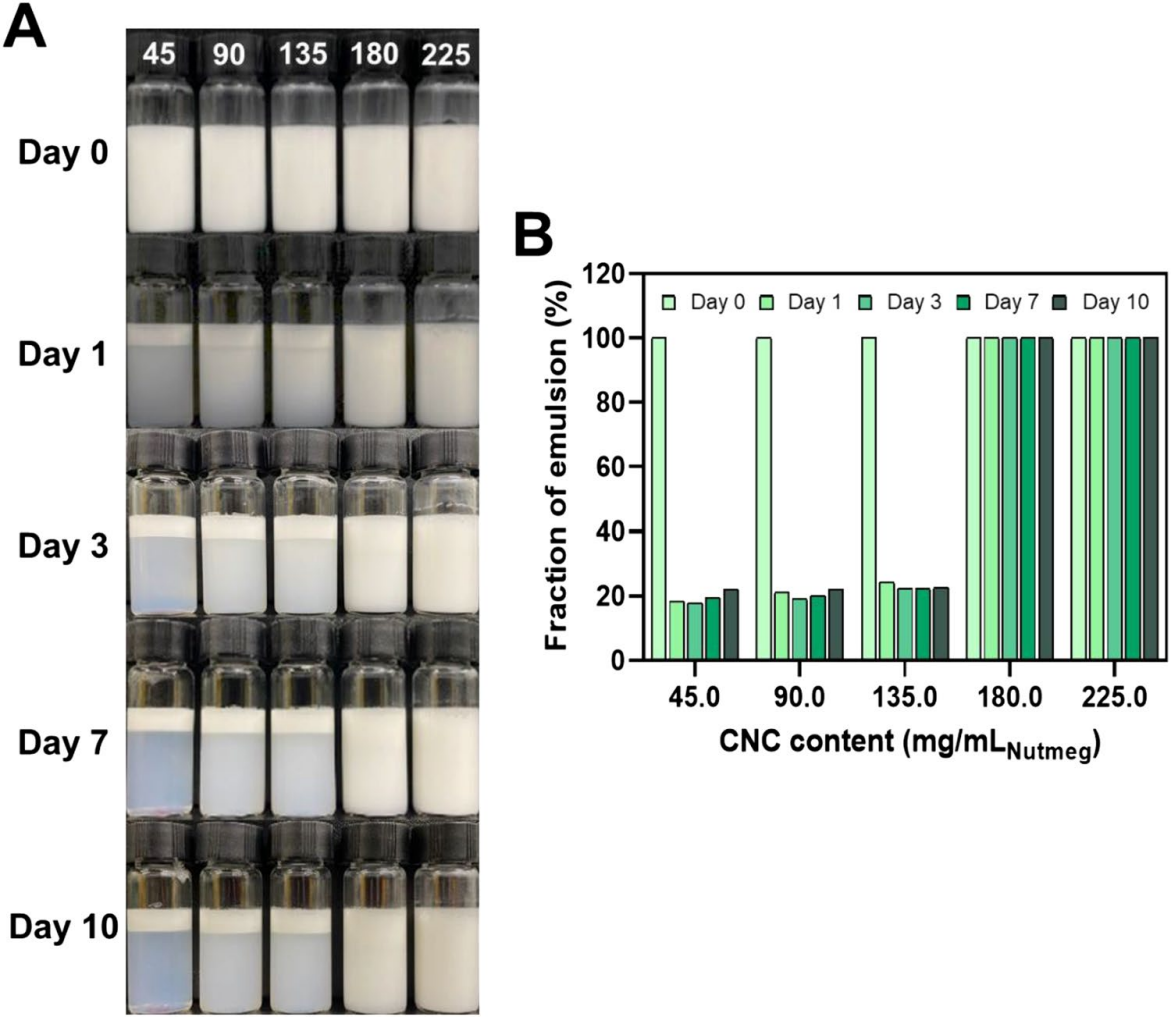
Phase separation of CNC/nutmeg PE solutions at different CNC content and storage time. (**A**) Digital images of PE solution and (**B**) the fraction of emulsion stored for 10 days.

**Figure 4:**
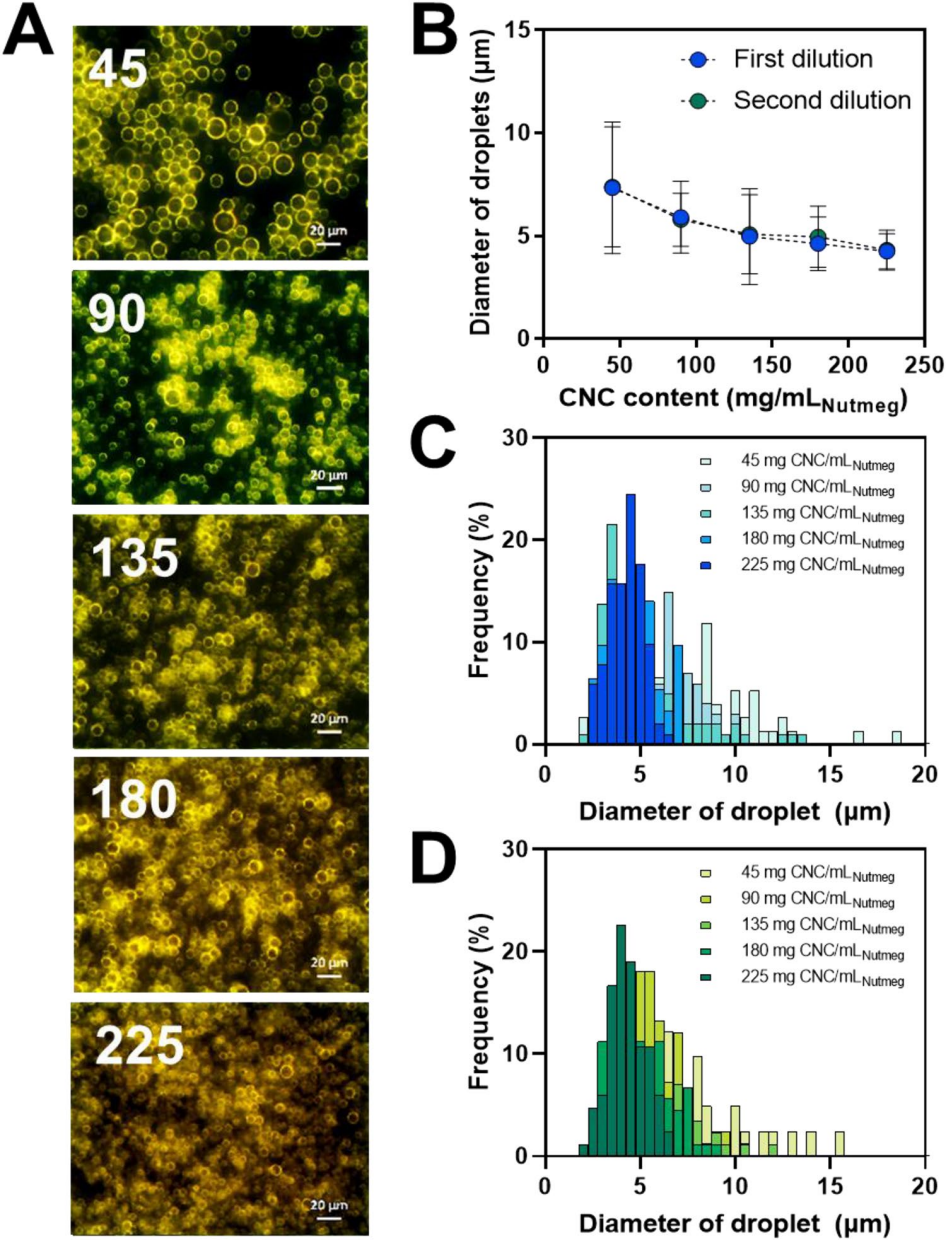
CNC/nutmeg PEs at different CNC contents. (**A**) Dark-field microscopic images of PE, (**B**) size of emulsion droplets and (**C**,**D**) size distribution of emulsion droplets. (**C**) First dilution and (**D**) second dilution. N = 300, error bar = SD.

**Figure 5:**
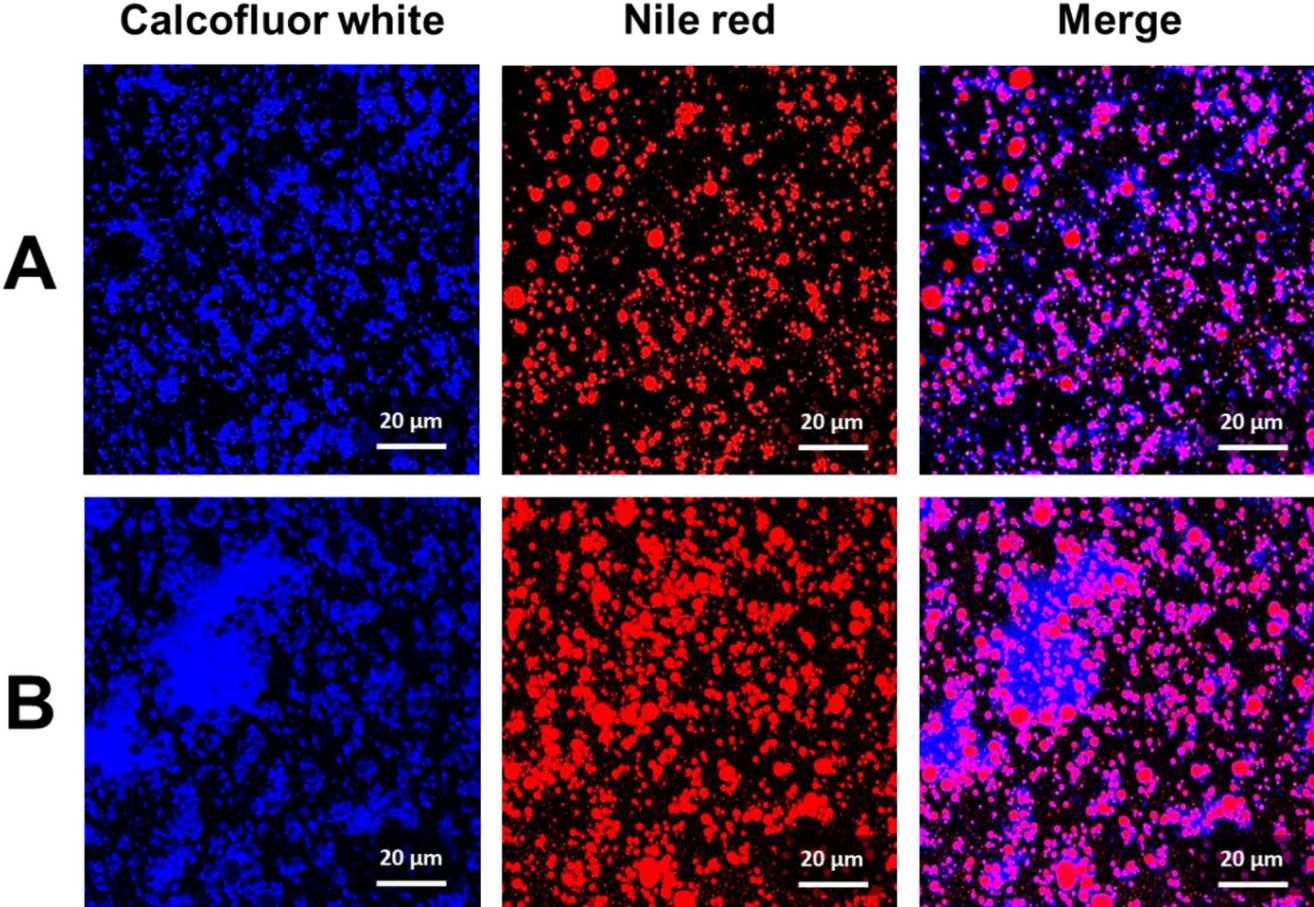
Confocal microscopic images of (**A**) CNC/massoia PEs (CNC 270 mg/mL_massoia_) and (**B**) CNC/nutmeg PEs (CNC 180 mg/mL_nutmeg_).

The stability of PEs can be evaluated by observing the phase separation of the emulsion solution and the change in emulsion fraction for long-term storage at different CNC contents (Fig. 1). Phase separation of the emulsion solution was observed at 135 and 180 mg of CNC/mL_massoia_ PEs. It resulted from insufficient CNC content for emulsifying the oils. In contrast, 270 mg of CNC/mL_massoia_ or more formed a stable emulsion state without phase separation for 10 days (Fig. 1A). The fraction of emulsion decreased after 10 days to approximately 20% for 135 and 180 mg CNC/mL_massoia_ PEs, but no big change was shown for 270 mg CNC/mL_massoia_ and more content (Fig. 1B). Microscopic images showed a shell structure of PEs due to the densely packed CNCs at the surface (Fig. 2A). Typically, PE size decreased as CNC content increased. The PEs ranged in size from 3 to 15 μm and the size distribution of PEs prepared at 135 and 180 mg CNC/mL_massoia_ was wider than that of the other PEs (Fig. 2B–D). Colloidal stability of nutmeg EO PEs was achieved at a lower CNC content compared with massoia PEs (Fig. 3A). Nutmeg EO PEs were stable at 180 and 225 mg of CNC/mL_nutmeg_ after storage for 10 days, while the emulsion fraction decreased to approximately 20% after storage for 1 day due to the phase separation of the emulsion at 45, 90, and 135 mg of CNC/mL_nutmeg_ (Fig. 3B). Microscopic images revealed a CNC shell structure at the surface of the PEs (Fig. 4A). The PEs ranged in size from 4 to 10 μm and the size decreased as the CNC content increased (Fig. 4B–D). The massoia and nutmeg EOs encapsulated in PEs was imaged by confocal microscopy (Fig. 5). Blue fluorescence represented CNCs stained with Calcofluor white, and red fluorescence represented EOs stained with Nile red. Both massoia and nutmeg EOs were encapsulated in the CNC PEs (Fig. 5A,B). The CNCs formed a shell structure at the surface of the PEs and the EOs were contained inside the shells. The densely packed CNC shells formed a rigid structure, reducing the aggregation of particles and maintaining colloidal stability for an extended period.

### Larvicidal activity of CNC-stabilized PE of massoia and nutmeg Eos

The larvicidal effect of CNC/massoia PEs and CNC/nutmeg PEs against *Ae. albopictus* are shown in Table 4. Larvicidal activities of CNC/massoia PE and CNC/nutmeg were higher than those of crude massoia and nutmeg EOs. Larvicidal activities of CNC/massoia PE, CNC/nutmeg PE, crude massoia EO, and crude nutmeg EO were 100%, 100%, 97.5% and 85.0% at 50 μg/mL, respectively. Mortality of *Ae. albopictus* treated with CNC PE only was 0%.

**Table 4:** Larvicidal activity of CNC-stabilized PEs of massoia and nutmeg EOs against *Ae. albopictus.*

Test materials	Mortality (%, Mean ± SE^a^, N^b^ = 4)			
	Concentration (μg/mL)			
	100	50	25	12.5
Massoia EO	100a^c^	97.5 ± 2.5a	37.5 ± 4.8bc	-
CNC/massoia PE	100a	100a	65.0 ± 8.7ab	12.5 ± 4.8a
Nutmeg EO	90.0 ± 4.1b	85.0 ± 9.6a	12.5 ± 2.5 cd	0b
CNC/nutmeg PE	100a	100a	82.5 ± 8.5a	12.5 ± 4.8a
Control (CNC PE only)	0c	0b	0d	0b
	F _4,15_ = 576	F _4,15_ = 95.36	F _4,15_ = 33.83	F _3,12_ = 4.55
	*P* < 0.0001	*P* < 0.0001	*P* < 0.0001	*P* = 0.024

^a^Standard error of the mean; ^b^Replication number; ^c^Means within a column followed by the same letters are not significantly different (Scheffe’s test).

## Discussion

Massoia and nutmeg EOs exhibited larvicidal activity against the Asian tiger mosquito, *Ae. albopictus*. The massoia tree is native to the rainforests of Indonesia and Papua New Guinea^22^. Its wood and bark are used in perfumes, food, and traditional medicine^22–24^. Biological activities of massoia EO, such as immunomodulatory effects, antifungal and phytotoxic activity have been reported^22,24,25^. Nutmeg is indigenous to the Moluccas and Banda islands in the South Pacific, and is now cultivated in several tropical regions^26^. Used as a traditional medicine for a long time, many pharmacological activities of nutmeg extracts and EOs have been documented^26–30^. Park et al.^31^ and Du et al.^32^ reported that nutmeg EO exhibited insecticidal activity against the mushroom sciarid fly *Lycoriella ingenua* and cigarette beetle *Lasioderma serricorne*. Although diverse biological activities of massoia and nutmeg EOs have been documented, there have been no reports on larvicidal activity of massoia EO especially against mosquito species including *Ae. albopictus*.

Rali et al.^33^ reported that the most abundant component of massoia EO was C10 massoia lactone (64.8%) followed by C12 massoia lactone (17.4%), benzyl benzoate (13.4%), *β*-bisabolene (1.4%), linalool (0.9%), and borneol (0.7%). Our chemical analysis of massoia EO was similar to that of a previous study^33^. Composition rates of C10 and C12 massoia lactones, benzyl benzoate, and benzyl salicylate were 79.97%, 10.75%, 6.55%, and 1.13%, respectively, but linalool and borneol were not detected in this study. Twenty-five compounds were identified as constituents of nutmeg EO in a previous study^34^, with the most abundant compound *α*-pinene (22.0%) followed by *β*-pinene (21.5%), sabinene (15.4%), myristicin (9.4%), terpinen-4-ol (5.7%), limonene (3.9%), myrcene (1.9%), *γ-*terpinene (1.8%), and *α*-thujene (1.2%). The contributions of other constituents were less than 1%. Fourteen compounds were found in nutmeg EO, with sabinene (50.44%) the most abundant. Detectable levels of *α*-thujene, camphene, *β*-pinene, *δ*-3-carene, *p*-cymene, linalool, borneol, *α*-terpineol, geraniol, bornyl acetate, eugenol, *α*-terpinyl acetate, neryl acetate, and methyl myristate were reported in a previous study^34^ but not in this study. Isoeugenol and elemicin were identified in this study but not in a previous study^34^. Harvest date, cultivation area, storage period, climate, and extraction method affected EO’s constituents and their ratios^35,36^. A chiral column could identify the enantiomers of two compounds of nutmeg EO, *α*-pinene and limonene, in this study. The composition ratio of ( +)-limonene was higher than that of (-)-limonene, but vice versa in *α*-pinene.

Larvicidal effect of EO constituents against *Ae. albopictus* have been documented in previous studies^37–42^. Lee et al.^40^ reported that the LC_50_ values of ( +)-*α*-pinene, (-)-*α*-pinene, myrcene, and ( +)-terpinen-4-ol against *Ae. albopictus* were 55.65, 28.61, and 35.98 mg/L, respectively. However, the values for ( +)-*α*-pinene, (-)-*α*-pinene, myrcene, and ( +)-terpinen-4-ol were > 100 μg/mL in this study. The larvicidal activities of ( +)-limonene, *α*-terpinene, *α*-phellendrene, and *γ*-terpinene reported in previous study^40^ were slightly stronger than those in this study, but the larvicidal activity of terpinolene was slightly weaker than that of this study. bin Jantan et al.^37^ reported that the LC_50_ values of benzayl salicylate, benzyl benzoate, and safrole were 5.5, 6.5, and 28.0 μg/mL, respectively. Larvicidal activity of benzyl salicylate was weaker than that of this study, but larvicidal activities of benzyl benzoate and safrole were slightly stronger. A previous research effort^37^ and this study discovered different larvicidal activities associated with eugenol and methyleugenol. Larvicidal activity of eugenol was stronger than that of methyleugenol in a previous study^37^ but the relationship was reversed in this study. In a report by Seo et al.^41^ the insecticidal activities of myristicin and elemicin were similar to those of this study. The differences in larvicidal activities of EOs constituents against *Ae. albopictus* may be attributable to the methodological differences, as bin Janten et al.^37^ used fourth instar larvae for larvicidal tests and ethanol as a solvent for EOs, unlike the method of this study.

C10 and C12 massoia lactones are reportedly the main constituents of massoia EO^23,33^, and they possess an *α-β*-unsaturated *δ*-lactone moiety. Massoia lactone groups have been reported to show biological activities, including antimicrobial, cytotoxic, anti-inflammatory and phytotoxic activities^22,24,43,44^. However, larvicidal activity against *Ae. albopictus* has not been reported for C10 and C12 massoia lactones.

Differences in the chemical structure of C10 and C12 massoia lactones include the aliphatic chain length at the C6 position, and this may be responsible for the difference in larvicidal activity against *Ae. albopictus*. Previous studies indicated that the chain length of compounds with similar chemical structures can play an important role in insecticidal and nematicidal activity^45,46^. Hammond and Kubo^45^ evaluated the larvicidal activity of alkanols with C_1_-C_20_ chain lengths against the mosquito *Culiseta incidens*. They found that larvicidal activities of dodecanol, tridecanol, and undecanol, with chain lengths of 12, 13, and 11, respectively, were stronger than those of alkanols with other chain lengths. Seo et al.^46^ also reported that an optimal chain length of aliphatic compounds was necessary for nematicidal activity against the pine wood nematode *Bursaphelenchus xylophilu.* Nematicidal activities of aliphatic compounds with a C_9_-C_11_ chain length were stronger than those of other aliphatic compounds with other chain lengths. Larvicidal activities of eugenol and its derivatives, such as methyleugenol, isoeugenol, and methylisoeugenol, showed that the methoxy group and a double-bond position affected activity. Larvicidal activities of eugenol and methyl eugenol with an allyl group were stronger than those of isoeugenol and methylisoeugenol with a propenyl group, and larvicidal activities of methyl eugenol and methylisoeugenol with two methoxy groups at a benzene ring were stronger than those of eugenol and isoeugenol with one methoxy group. Bhardwaj et al.^47^ reported that larvicidal activity of methyl eugenol was stronger than that of eugenol against the tobacco armyworm, *Spodopter litura*. Methyleugenol exhibited strong contact toxicity against the cigarette beetle, *L. serricorne* compared with methylisoeugenol^32^.

The insolubility of plant EOs in water requires emulsion-based formula for the practical use of plant EOs as larvicides against mosquitoes. Emulsions generally comprise small spherical droplets of two liquids stabilized with surfactants or surface-active polymers^48^, and are used widely in the pesticide industry^20^. However, conventional emulsions made with synthetic emulsifiers such as alkylphenol ethoxylates and organic-phase emulsifiers such as toluene and xylene have many drawbacks^20^, requiring the development of environmentally friendly emulsions, and PEs stabilized by solid particles are considered alternatives for conventional emulsions^49–52^. CNCs are appropriate solid particles for manufacturing PEs due to their high aspect ratio of crystalline fibrils and amphiphilicity^48,53^. In this study, CNC-stabilized PEs of massoia and nutmeg EOs were made and their physiological properties and larvicidal activity were investigated. The PEs exhibited visual differences depending on CNC content after storage for 24 h (Figs. 1A, 3A). This study shows that a critical concentration of CNCs is required to cover EOs completely and ensure the long-term stabilization of CNC-based PEs. The critical concentrations of CNC/mL EOs for massoia and nutmeg EOs were approximately 270 mg and 180 mg, respectively. Shin et al.^19^ reported a critical concentration of CNC/mL EOs for thyme white EO that was close to 135 mg. This and previous studies indicated that the effective concentration (CNC/mL) for EOs varied according to the EOs. Particle-size study of CNC-based PEs have also produced valuable information to determine the appropriate concentration of EOs. The mean diameter of droplets and distribution of the sizes of emulsions at different contents of CNCs indicated that 270 mg of CNC/mL of EO was the most effective concentration for massoia EO. In the case of nutmeg EO, 180 mg and 225 mg of CNC/mL of EO had a smaller emulsion size and narrower distribution than the other PEs. Based on this result, 270 mg and 180 mg of CNC/mL for massoia and nutmeg EOs were finally chosen to evaluate the larvicidal activity, respectively.

The results of this study demonstrate that CNC-based PEs of massoia and nutmeg EOs as promising larvicides which can be applied in field, since the larvicidal activies of CNC/massoia and CNC/nutmeg PEs were stronger than those of crude massoia and nutmeg EOs. Also, CNC-based PEs have showed stability for at least 10 days after the production, with improved solubility of massoia and nutmeg EOs in water. Another advantage of CNC-based PEs may be controlled release of EOs, as EOs and their constituents easily evaporate when treated in water^15,41^. However, further studies about proper dilution of the formulation, safety to non-target organisms, radiation and temperature effect after application and other factors such as costs are required for practical application of CNC-based PEs of massoia and nutmeg EOs.

## Methods

### Plant essential oils

The information about EOs used in this experiment are shown in Table 1. Blue cypress, Hinoki, Texas cedar, Japanese cedar, Golden-lotus, Cubeb and massoia EOs were purchased from Oshadhi Ltd. (Cambridge, UK). Fir needle, Spice, spruce and Nutmeg EOs were purchased from Jin Aromatics (Anyang, Republic of Korea).

### Chemicals

Chemicals used in chemical analysis of the EOs and the components of EOs used in larvicidal activity test are listed below. Eugenol and methyl eugenol were used in larvicidal activity test based on the structure–activity relationship of eugenol derivatives against *Ae. aegypti*^54^. Eicosane (99% purity), heptadecane (99%), isoeugenol (98%), ( +)-limonene (97%), methyl eugenol (99%), myrcene (95%), myristicin (99%), nonadecane (99%), nonane (99%), octadecane (99%), octane (98%), *S*-( −)-*α*-pinene (99%), safrole (97%), temephos (95.6%), and tridecane (99%) were purchased from Sigma-Aldrich (Milwaukee, WI, USA), and eugenol (99%), *α*-terpinene (85%), *γ*-terpinene (97%), and terpinen-4-ol (97%) from Fluka (Buchs, Switzerland). Benzyl benzoate (99%), benzyl salicylate (95%), docosane (99%), heneicosane (99.5%), heptacosane (97%), hexacosane (99%), ( −)-limonene (95%), methyl isoeugenol (98%), nonacosane (98%), octacosane (98%), pentacosane (99%), α-phellandrene (65%), *R*-( +)-*α*-Pinene (> 95%), tetracosane (99%), and tricosane (95%) were obtained from Tokyo Chemical Industry (Tokyo, Japan). Decane (99.5%), dodecane (99%), hexadecane (97%), pentadecane (97%), tetradecane (99%), and undecane (99%) were purchased from Wako (Osaka, Japan). Elemicin (99%) was supplied by Santa Cruz Biotechnology (Dallas, TX, USA). C10 and C12 massoia lactones were isolated from massoia EO. Silica gel was purchased from Merck (0.006 − 0.2 mm, Kenilworth, NJ, USA).

### Insects

Cultures of *Ae. albopictus* were supplied from Korean Disease Control and Prevention Agency (Cheongju, Republic of Korea). They were reared in an insectary at 26 ± 1 °C and a relative humidity of 60 ± 5% under a 16:8 h light/dark cycle. We supplied the larva with a sterilized diet composed of 40-mesh chick chow powder and yeast (4:1). All procedures involving the use of animals were performed in compliance with the ARRIVE guidelines for animal studies. A live mouse was provided as a blood-meal source, using a method approved by the Institutional Animal Care and Use Committee (approval no. SNU-190418–1-2; title: Providing rodents for blood-feeding mosquitoes to assess the effectiveness of insecticides against mosquitoes).

### Larvicidal activity test

Each EO or compound (50 mg) was dissolved in 5 mL of acetone (10,000 μg/mL) to make a stock solution. For each EO or compound solution, up to 6 more concentrations (5,000, 2,500, 1,250, 625, 312.5 and 156.25 μg/mL) were prepared by serial dilution. Likewise, 0.5 g of CNC-stabilized PEs of massoia and nutmeg EOs (100 mg/g) were dissolved in 5 mL of double distilled water (10,000 μg/mL) to make a stock solution. For each CNC-stabilized PEs solution, up to 3 more concentrations (5,000, 2,500 and 1,250 μg/mL) were prepared by serial dilution. Each test solution (1 mL) was suspended in 99 mL of water in 6.5-oz paper cups. 10 early-third-instar larvae of *Ae. albopictus* were used for each treatment^55^. 1 mL of acetone and CNC-stabilized PE without EOs was used for negative control, and temephos was used as a positive control. Treated and control larvae were kept at the same condition for maintenance. Larval mortality was recorded 48 h post-treatment.

### Gas chromatography

An Agilent 7890 N (Santa Clara, CA, USA) gas chromatograph (GC) with a flame ionization detector was used to analyze the chemical composition of massoia and nutmeg EOs. We used DB-5MS columns (length: 30 m; internal diameter: 0.25 mm; film thickness: 0.1 μm) and HP-INNOWAX columns (length: 30 m; internal diameter: 0.25 mm; film thickness: 25 μm) (J&W Scientific, Folsom, CA, USA) to measure the retention time and retention index (RI) of EO components. The RI of each peak was determined by calculating the relationship to a homologous series of n-alkanes (C_8_-C_29_; DB-5MS, HP-INNOWax) under the same GC operating conditions^56^. The oven was programmed to be isothermal at 40 °C for 1 min then, heat to 250 °C at the rate of 6 °C/min and hold at this temperature for 4 min. Nitrogen was used as the carrier gas at a rate of 1 mL/min. To determine the configurations of limonene and *α*-pinene, a Beta DEX 120 chiral column (length: 30 m; internal diameter: 0.25 mm; film thickness: 0.25 μm) (Supelco, Bellefonte, PA, USA) was used. The oven was programmed to be isothermal at 40 °C for 5 min then, heat to 230 °C at 6 °C/ min and hold at this temperature for 10 min. Nitrogen flowing at 1 mL/min was used as a carrier gas.

### Gas chromatography − mass spectrometry

Agilent 7890B GC and an Agilent 5977B MSD mass spectrometer were used to identify the chemical components of massoia and nutmeg EOs. An HP-5MS column (length: 30 m; internal diameter: 0.25 mm; film thickness: 0.25 μm) (J&W Scientific, Folsom, CA, USA) was used, and the flow rate of the carrier gas (helium) was 1.0 mL/min. The oven was programmed to be isothermal at 40 °C for 5 min then, heat to 250 °C at the rate of 6 °C/min and hold at this temperature for 5 min. Ionization was obtained by electron impact (70 eV, source temperature of 230 °C), and the scan range was 41 − 400 amu. Most EO components were identified by comparing the mass spectra of each peak with those of standard compounds obtained from the National Institute of Standards and Technology mass spectrometry library.

### Isolation of C10 and C12 massoia lactones

Commercially unavailable C10 and C12 massoia lactones were obtained from massoia EO via a bioassay-guided isolation process. C10 massoia lactone (1.8 g) and C12 massoia lactone (87 mg) were isolated from 9.9 g of massoia EO on SiO_2_ gel using column chromatography with hexane/diethyl ether solution (100:0 → 0:100) as the mobile phase. The fractions were combined and the solvent was removed using rotary a evaporator at 35 ℃. The concentrates were then used for bioassays and further purification of the active compounds. Pure C10 massoia lactone and C12 massoia lactone were isolated from hexane/diethyl ether fractions of 70:30 and 93:7, respectively. ^1^H and ^13^C NMR spectra were obtained on a Bruker Avance 600 NMR spectrometer (600 MHz for ^1^H spectra and 150 MHz for ^13^C spectra, Billerica, MA, USA) using CDCl_3_ as the solvent at the National Instrumentation Center for Environmental Management, College of Agricultural and Life Science, Seoul National University (Seoul, Republic of Korea).

### Preparation of sulfated-CNC

Sulfated CNCs were prepared from 10 g of cotton pulp (Whatman, Grade 2, Kent, UK) and 100 mL of 60% (w/w) sulfuric acid solution (Junsei Chemical Co. Ltd., 95% w/w purity, Tokyo, Japan)^19,57^. Finely cut filter paper was mixed with a sulfuric acid solution and stirred at 45 °C for 60 min. The hydrolyzed CNC solution was centrifuged with de-ionized water at 6,000 rpm for 15 min. The well-dispersed CNC solution was dialyzed for 7 days using a cellulose dialysis membrane (MWCO 12–14 kDa, Spectra/Por, Breda, the Netherlands).

### Preparation of CNC-stabilized massoia and nutmeg PEs

Massoia and nutmeg EO PEs were mixed with the CNC solution at a 10% ratio and tip-sonicated at a 60% amplitude for 1 min using an ultrasonic processor (VCX 130, Sonics & Materials Inc., Newtown, CT, USA). The CNC contents were fixed at 135, 180, 270, 360 and 450 mg per 1 mL of massoia EO and 45, 90, 135, 180 and 225 mg per 1 mL of nutmeg EO. Each PE solution was stored for 10 days at room temperature to allow for observation of phase separation of the solution, and the height of phase separation was measured using ImageJ software (1.52a, National Institutes of Health, Bethesda, MA, USA). The fraction of each emulsion was calculated by dividing the height of the creaming layer by the height of the total solution. The droplet shape and the size of the PEs were observed by a polarized light microscope (LV100, Nikon, Tokyo, Japan) in dark-field mode. The first droplet size was measured after diluting 20 times, and the second droplet size was measured after diluting the solution diluted 20 times again 2 times (40 times)^58^. Size distribution of the PE droplets was characterized using ImageJ. The CNCs were stained with Calcofluor white dye (Sigma-Aldrich, St. Louis, USA) while massoia and nutmeg EOs were stained with Nile red (sigma-Aldrich, St. Louis, USA) for confocal laser scanning microscopy (SP8 X, Leica, Wetzlar, Germany).

### Statistical analysis

Mortality data were transformed to arcsine square root values for one-way analysis of variance (ANOVA). Treatment means were compared and separated by Scheffe’s test. All statistical analysis was carried out in IBM SPSS Statistics 26.0 (2019).

## Conclusion

Our results showed that CNC-stabilized PEs of massoia and nutmeg EOs were stable and dispersed easily in water. In addition, larvicidal activities of the CNC-stabilized PEs of massoia and nutmeg EOs were stronger than those of crude EOs. This indicates that a CNC-stabilized PE is a suitable formulation for EOs as a larvicide against mosquitoes in field applications.
